# Three-year risk of cardiovascular disease among intensive care patients with acute kidney injury: a population-based cohort study

**DOI:** 10.1186/s13054-014-0492-2

**Published:** 2014-10-14

**Authors:** Henrik Gammelager, Christian Fynbo Christiansen, Martin Berg Johansen, Else Tønnesen, Bente Jespersen, Henrik Toft Sørensen

**Affiliations:** Department of Clinical Epidemiology, Aarhus University Hospital, Olof Palmes Allé 43-45, Aarhus, DK-8200 Denmark; Departments of Anesthesiology and Intensive Care Medicine, Aarhus University Hospital, Brendstrupgårdsvej 100, Aarhus, DK-8200 Denmark; Department of Renal Medicine, Aarhus University Hospital, Skejley Sygehus, Aarhus, DK-8200 Denmark

## Abstract

**Introduction:**

Acute kidney injury (AKI) is common among intensive care unit (ICU) patients, but follow-up data on subsequent risk of cardiovascular disease remain sparse. We examined the impact of AKI on three-year risk of first-time heart failure, myocardial infarction (MI), and stroke among ICU patients surviving to hospital discharge, and whether this risk is modified by renal recovery before hospital discharge.

**Methods:**

We used population-based medical registries to identify all adult patients admitted to an ICU in Northern Denmark between 2005 and 2010 who survived to hospital discharge and who had no previous or concurrent diagnosis of heart failure, MI, or stroke. AKI was defined according to the creatinine criteria in the Kidney Disease Improving Global Outcomes classification. We computed the three-year cumulative risk of hospitalization with heart failure, MI, and stroke for patients with and without AKI and the hazard ratios (HRs), using a Cox model adjusted for potential confounders.

**Results:**

Among 21,556 ICU patients surviving to hospital discharge, 4,792 (22.2%) had an AKI episode. Three-year cumulative risk of heart failure was 2.2% in patients without AKI, 5.0% for AKI stage 1, and 5.0% for stages 2 to 3. The corresponding adjusted HRs were 1.33 (95% confidence interval (CI), 1.06 to 1.66) for patients with AKI stage 1 and 1.45 (95% CI, 1.14 to 1.84) for AKI stages 2 to 3, compared to patients without AKI. The three-year cumulative MI risk was 1.0% for patients without AKI, 1.8% for patients with AKI stage 1 and 2.3% for patients with AKI stages 2 to 3. The adjusted HR for MI was 1.04 (95% CI, 0.71 to 1.51) for patients with AKI stage 1 and 1.51 (95% CI, 1.05 to 2.18) for patients with AKI stages 2 to 3, compared with patients without AKI. We found no association between AKI and stroke. The increased risk of heart failure and MI persisted in patients with renal recovery before discharge, although it was less pronounced than in patients without renal recovery.

**Conclusions:**

ICU patients surviving any stage of AKI are at increased three-year risk of heart failure, but not stroke. Only AKI stages 2 to 3 are associated with increased MI risk.

**Electronic supplementary material:**

The online version of this article (doi:10.1186/s13054-014-0492-2) contains supplementary material, which is available to authorized users.

## Introduction

Acute kidney injury (AKI), which occurs in 22% to 67% of intensive care unit (ICU) patients [1-5], is associated with increased risk of both chronic kidney disease (CKD) and death [1-8].

AKI also may have long-term adverse cardiovascular effects [9]. The potentially increased risk of cardiovascular disease following AKI might be mediated through chronic renal impairment, a well-known risk factor of cardiovascular disease [10]. In addition, animal studies have shown that AKI causes a systematic inflammatory response and activation of the renin-angiotensin system, subsequently promoting apoptosis and interstitial/perivascular fibrosis in the myocardium, and ultimately cardiac dysfunction [11,12].

These findings are supported by a few cohort studies in humans, which reported that patients with AKI as a complication to myocardial infarction (MI), coronary intervention, or heart failure (HF) have an increased risk of subsequent HF and MI [13-18]. AKI during hospitalization also increases the risk of subsequent HF in patients infected with human immunodeficiency virus (HIV) [19]. However, it is not known whether AKI has similar implications for incident HF and MI among ICU patients, among whom AKI is common [1-5]. Although it has been suggested that AKI may increase stroke risk [20], the potential association between AKI and long-term stroke risk has received little attention [13].

We therefore conducted a population-based cohort study to examine (1) the impact of AKI on three-year risk of first-time HF, MI, and stroke among ICU patients surviving to hospital discharge, and (2) whether recovery of renal function before hospital discharge modifies subsequent risk of these cardiovascular diseases.

## Methods

### Setting

We conducted this cohort study using population-based medical databases in Northern Denmark with approximately 1.15 million inhabitants.

The Danish health care system provides tax-funded health care to all Danish residents. The study region has 12 ICUs, eight at university hospitals (one cardiothoracic, one mixed cardiothoracic and multidisciplinary, one mixed neurosurgery and multidisciplinary, one neurosurgical, and four multidisciplinary) and four at regional hospitals (all multidisciplinary). The unique civil registration number assigned to all Danish residents allowed us to link Danish medical and administrative databases [21].

### Intensive care patients

We used the Danish National Registry of Patients (DNRP) covering all Danish hospitals to identify all adult residents of Northern Denmark (aged 15 years or older) who had a first-time ICU admission from 1 January 2005 to 31 December 2010 and who survived to hospital discharge. We required one year of residency in the study region to ensure availability of previous test results from the regional laboratory database. The study period was chosen based on availability of data. The DNRP includes data on all nonpsychiatric hospitalizations in Denmark since 1977. Since 1995, the DNRP also has included data from outpatient clinic visits, emergency room visits, and psychiatric units. The DNRP records patients’ civil registration number, treating hospital and department, dates of admission and discharge, type of admission (emergency vs. planned), surgical and other major procedures performed, ICU admissions and treatments, and one primary and up to 19 secondary discharge diagnoses assigned by the discharging physician. According to Danish guidelines, the primary diagnosis is the main reason for the hospital admission. Since 1994, discharge diagnoses have been coded using the *International Classification of Diseases*, *10*^*th*^*revision* (ICD-10) [22]. We used the primary ICD-10 diagnosis for the current hospitalization to categorize patients into one of the following eight major disease groups: infectious disease, endocrine disease, cardiovascular disease, respiratory disease, gastrointestinal or liver disease, cancer, trauma or poisoning, and other. In addition, we categorized patients into five groups according to surgical status: no surgery, acute cardiac surgery, acute noncardiac surgery, elective cardiac surgery, and elective noncardiac surgery [6].

### Acute kidney injury

We used the population-based laboratory database that covers the study region to identify occurrences of AKI. The laboratory database contains test results from all inpatient stays, outpatient clinic visits, and visits to general practitioners [23]. We classified AKI according to the serum creatinine criteria in the Kidney Disease Improving Global Outcome (KDIGO) AKI classification (Table 1) [24]. We searched the laboratory database for the highest plasma creatinine (equivalent to serum creatinine [25]) value from ICU admission until hospital discharge to determine the highest level of AKI. All other ICU patients were classified as being ‘without AKI’. We defined the baseline creatinine level as the most recent creatinine measurement from an outpatient clinic or general practitioner visit in the period from one year to seven days before the current hospitalization [26]. Plasma creatinine assessments later than seven days before the current hospitalization were not considered, because acute renal impairment may precede the admission. For patients without CKD who lacked a baseline creatinine measurement, we estimated baseline creatinine using the four-variable version of the Modification of Diet in Renal Disease (MDRD) equation based on age, race, and gender, as recommended by the Second International Consensus Conference of the Acute Dialysis Quality Initiative (ADQI) Group [27]. Patients previously treated with chronic dialysis or hemofiltration, those with a previous kidney transplant, and those lacking information on plasma creatinine levels following ICU admission were excluded from the study.

**Table 1:** **KDIGO serum creatinine classification of acute kidney injury** [24]

**Stage**	**Serum creatinine criteria**
1	1.5-1.9 times baseline
	OR
	≥ 26.5 5 μmol/l (0.3 mg/dl) increase within 48 hours^a^
2	2.0-2.9 times baseline
3	≥ 3.0 times baseline
	OR
	Increase in serum creatinine to ≥ 354 μmol/l (4.0 mg/dl)^b^
	OR
	Initiation of renal replacement therapy

^a^We defined 48 hours as two calendar days; ^b^together with fulfillment of any of the other criteria for acute kidney injury. KDIGO, Kidney Disease Improving Global Outcomes.

### Heart failure, myocardial infarction, and stroke

Study outcomes were any hospital admission with a diagnosis of HF, MI, or stroke (ischemic and hemorrhagic cerebral stroke) registered in the DNRP subsequent to the current hospital admission up to three years after hospital discharge. In order to exclude prevalent cases, we omitted all patients with any previous diagnosis of HF, MI, or stroke up to 10 years prior to or during the admission.

### Renal recovery at hospital discharge

Patients were considered to have achieved renal recovery at hospital discharge if they did not receive any type of dialysis or hemofiltration treatment up to seven days before hospital discharge and their last plasma creatinine measurement before discharge was less than 50% above their baseline level, that is, they no longer fulfilled the criteria for AKI [28].

### Covariates

We obtained data on covariates potentially associated with both AKI and the study outcomes from the DNRP [22], the laboratory database [23], and the Aarhus University Prescription Database covering all community pharmacies in the study region [29].

Data on comorbidities were primarily obtained from the DNRP, including inpatient or outpatient clinic diagnoses up to 10 years before the current hospitalization. Because many patients with diabetes are treated outside hospitals, we also defined patients as having diabetes if they had a hemoglobin A1c level at or above 6.5% [30] in the laboratory database up to one year before the current admission or had any prescription for an antidiabetic drug (insulin or oral antidiabetic agent) in the Aarhus University Prescription Database up to five years before the current admission. Diabetes patients who were identified solely by a prescription of metformin and who had coexisting polycystic ovary syndrome were classified as not having diabetes. The Aarhus University Prescription Database contains information on all filled drug prescriptions in the study region. Variables included in this database are patients’ civil registration number, type of drug according to the Anatomic Therapeutic Chemical Classification System (ATC), dosage, and prescription date [29]. Information on CKD was obtained from the laboratory database, defined as estimated glomerular filtration rate (eGFR) below 60 ml/min per 1.73 m^2^ using the four-variable MDRD equation (stage 3 or higher CKD according to National Kidney Foundation guidelines) [24]. We used the most recent plasma creatinine measurement from an outpatient clinic or general practitioner visit within one year to seven days before the current hospitalization to compute the eGFR [26]. Patients lacking a plasma creatinine measurement were considered as not having CKD. We also retrieved information on preadmission drug use, defined as a prescription filled from 90 days before the current hospitalization until hospital admission [31]. Information was obtained for all drugs listed in Table 2.

All relevant codes used to retrieve data from the DNRP, the laboratory database, and the Aarhus University Prescription Database are provided in Additional file 1.

### Statistical analyses

For each outcome, we followed patients from date of hospital discharge for up to three years or until first-time hospital admission with a study outcome of death or the end of follow-up on 31 December 2011, whichever came first.

We used the cumulative incidence method to plot and compute the crude three-year cumulative risk of admission with HF, MI, or stroke for patients with AKI stage 1, AKI stages 2 to 3, and those without AKI, taking death into account as a competing risk [32]. Using hazard ratios (HRs) computed with Cox regression models, we compared the risk of each outcome for patients with AKI stage 1 and AKI stages 2 to 3 with that of patients without AKI [33]. In multivariate analyses we adjusted for the following potential confounders: age, gender, other heart disease, other cerebrovascular disease, hypertension, peripheral vascular disease, diabetes, CKD, cancer, surgical status at ICU admission, primary diagnosis during current hospitalization, and preadmission use of any drug listed in Table 2. To examine the influence of renal recovery on subsequent cardiovascular disease risk, we stratified the analysis by renal recovery status at hospital discharge. All estimates were reported with 95% confidence intervals (CI).

**Table 2:** **Patient characteristics by AKI status among ICU patients surviving to hospital discharge, Northern Denmark, 2005 to 2010**

	**Without AKI**	**AKI stage 1**	**AKI stages 2-3**
	**n = 16,764**	**n = 2,666**	**n = 2,126**
**Age**			
Age, median (IQR)	57 (39-69)	68 (58-77)	67 (56-75)
**Gender**			
Female	7,788 (46.5)	1,031 (38.7)	944 (44.4)
Male	8,976 (53.5)	1,635 (61.3)	1,182 (55.6)
**Comorbidity**			
Ischemic heart disease except MI^a^	1,863 (11.1)	513 (19.2)	244 (11.5)
Cerebrovascular disease except stroke^b^	537 (3.2)	124 (4.7)	86 (4.0)
Diabetes	1,490 (8.9)	460 (17.3)	433 (20.4)
Chronic kidney disease	1,063 (6.3)	502 (18.8)	338 (15.9)
Hypertension	2,216 (13.2)	664 (24.9)	511 (24.0)
Peripheral vascular disease	721 (4.3)	266 (10.0)	202 (9.5)
Cancer	2,282 (13.6)	522 (19.6)	375 (17.6)
No surgery	6,455 (38.5)	662 (24.8)	793 (37.3)
Surgery			
Acute cardiac surgery	195 (1.2)	88 (3.3)	59 (2.8)
Acute noncardiac surgery	5,558 (33.2)	834 (31.3)	764 (35.9)
Elective cardiac surgery	1,701 (10.1)	528 (19.8)	169 (7.9)
Elective noncardiac surgery	2,855 (17.0)	554 (20.8)	341 (16.0)
**Preadmission drug use**			
ACE inhibitors/AT2 antagonists	2,677 (16.0)	763 (28.6)	698 (32.8)
Beta blockers	2,318 (13.8)	667 (25.0)	487 (22.9)
Calcium channel antagonists	1,587 (9.5)	483 (18.1)	401 (18.9)
Acetylsalicylic acid	5,286 (31.5)	1,362 (51.1)	1,005 (47.3)
Diuretics	1,348 (8.0)	357 (13.4)	272 (12.8)
Nitrates	756 (4.5)	236 (8.9)	83 (3.9)
Statins	2,533 (15.1)	671 (25.2)	441 (20.7)
NSAIDs	2,486 (14.8)	430 (16.1)	436 (20.5)
**Primary diagnosis during current hospitalization**			
Infectious diseases	1,673 (10.0)	276 (10.4)	434 (20.4)
Endocrine diseases	302 (1.8)	69 (2.6)	99 (4.7)
Cardiovascular diseases	2,910 (17.4)	873 (32.7)	390 (18.3)
Respiratory diseases	741 (4.4)	118 (4.4)	108 (5.1)
Gastrointestinal or liver diseases	1,564 (9.3)	287 (10.8)	303 (14.3)
Cancer or other neoplasm	2,463 (14.7)	471 (17.7)	304 (14.3)
Trauma or poisoning	4,198 (25.0)	282 (10.6)	190 (8.9)
Other	2,913 (17.4)	290 (10.9)	298 (14.0)
**Laboratory information**			
Measured baseline creatinine	10,213 (60.9)	2,143 (80.4)	1,610 (75.7)
Maximum creatinine during admission,^c^ μmol/l, mean (IQR)	74 (62-88)	123 (101-149)	254 (180-397)
**ICU treatments**			
Mechanical ventilation	4,362 (26.0)	1,297 (48.6)	1,126 (53.0)
Inotropes/vasopressors	3,083 (18.4)	1,158 (43.4)	1,155 (52.9)
**Length of admission**			
In-hospital days^d^, median (IQR)	8 (3-15)	15 (10-28)	23 (12-45)

Values are expressed as number (percentage) unless otherwise indicated. ^a^Patients with a previous diagnosis of myocardial infarction were not included in the study; ^b^patients with a previous diagnosis of stroke were not included in the study; ^c^from ICU admission until hospital discharge; ^d^from hospital admission to hospital discharge. If date of discharge from one department and/or hospital and admission to another department and/or hospital was ≤1 calendar day, this was considered as one hospital admission. ACE, angiotensin-converting enzyme; AKI, acute kidney injury; AT2, angiotensin 2; ICU, intensive care unit; IQR interquartile range; n, number; NSAIDS, nonsteroidal anti-inflammatory drugs.

We conducted a sensitivity analysis to examine the potential influence of excluding patients without a plasma creatinine measurement for each outcome of interest. We imputed AKI levels for patient without a creatinine measurement using multiple imputation [34-36]. HRs for each outcome was calculated as the average HR of the five datasets, corrected for between- and within-imputation variation [34-36]. The imputation models included all variables in Table 2, the outcome of interest, and the Nelson Aalen estimator of the cumulative baseline hazard evaluated at the observed survival time [37].

The assumption of proportional hazards for all Cox regression models was assessed graphically using log(−log(survival probability))-plots and found valid. Analyses were performed using the statistical software package Stata, version 11.1 (StataCorp LP, College Station, TX, USA).

All data were obtained from Danish registries, which are generally available to researchers without ethics approval or informed consent [38,39]. The study was approved by the Danish Data Protection Agency (record number 2009-41-3987).

## Results

### Characteristics of the study population

The study population comprised 21,556 ICU patients who survived to hospital discharge, after excluding those with a previous kidney transplant or chronic dialysis treatment (n = 314), those with a previous or concurrent diagnosis of HF, MI, or stroke (n = 6,702), and those lacking a plasma creatinine measurement upon or after ICU admission (n = 1,846). Compared to patients with a plasma creatinine measurement, patients lacking this measurement were younger, had less comorbidity, and had a markedly shorter hospital stay (Additional file 2). Median duration of follow-up was 2.7 years for all three study outcomes.

We found that 4,792 (22.2%) of the 21,556 ICU patients had an episode of AKI; 2,666 (12.4%) with AKI stage 1 and 2,126 (9.9%) with AKI stages 2 to 3. Patients with AKI were older, more often male, and had a higher degree of comorbidity than other ICU patients. AKI patients also were more frequently users of cardiovascular drugs and nonsteroidal anti-inflammatory drugs (NSAIDs) (Table 2). The primary diagnosis during the current hospitalization was most frequently trauma and poisoning for patients without AKI (25.0%), cardiovascular disease for patients with AKI stage 1 (32.7%), and infectious disease for patients with AKI stages 2 to 3 (20.4%). Patients with AKI were more frequently treated with mechanical ventilation and inotropes/vasopressors and had longer hospital stays (Table 2). During follow-up, 2,569 (15.3%) died without AKI, 619 (23.3%) with AKI stage 1 and 558 (26.2%) with AKI stages 2 to 3.

### Risk of heart failure

**Figure 1:**
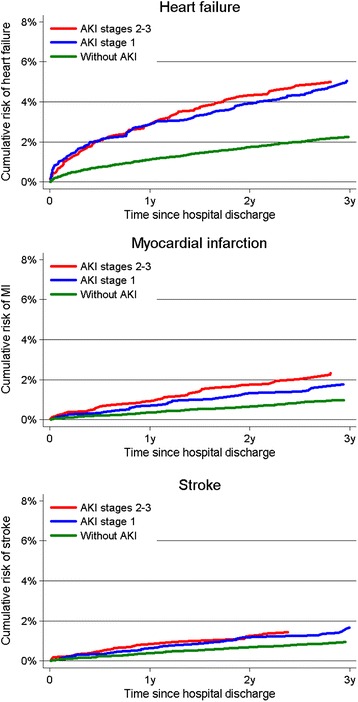
**Three-year crude cumulative incidence of heart failure, myocardial infarction, and stroke by AKI stage.** AKI, acute kidney injury.

During the three years following hospital discharge, 2.2% of patients without AKI, 5.0% of patients with AKI stage 1, and 5.0% of patients with AKI stages 2 to 3 were hospitalized with first-time HF (Figure 1 and Table 3). Compared to patients without AKI, the adjusted HR was 1.33 (95% CI, 1.06 to 1.66) for patients with AKI stage 1 and 1.45 (95% CI, 1.14 to 1.84) for patients with AKI stages 2 to 3 (Table 3).

**Table 3:** **Three-year risk of cardiovascular disease according to AKI stage, Northern Denmark, 2005 to 2010**

	**Without AKI**		**AKI stage 1**				**AKI stages 2-3**			
	**Events, n**	**Cumulative risk % (95% CI)**	**Events, n**	**Cumulative risk % (95% CI)**	**Unadjusted HR (95% CI)**	**Adjusted HR** ^**a**^ **(95% CI)**	**Events, n**	**Cumulative risk % (95% CI)**	**Unadjusted HR (95% CI)**	**Adjusted HR** ^**a**^ **(95% CI)**
HF	320	2.2 (2.0–2.5)	114	5.0 (4.2–6.0)	2.43 (1.96–3.01)	1.33 (1.06–1.66)	91	5.0 (4.0–6.1	2.53 (2.01–3.01)	1.45 (1.14–1.84)
MI	135	1.0 (0.8–1.2)	38	1.8 (1.3–2.4)	1.93 (1.35–2.76)	1.04 (0.71–1.51)	40	2.3 (1.7–3.1)	2.68 (1.88–3.82)	1.51 (1.05–2.18)
Stroke	131	0.9 (0.8–1.1)	35	1.7 (1.2–2.4)	1.83 (1.26–2.65)	1.10 (0.75–1.62)	26	1.4 (1.0–2.1)	1.79 (1.17–2.72)	1.07 (0.70–1.65)

^a^Adjusted for age, gender, other ischemic heart diseases, other cerebrovascular diseases, hypertension, peripheral vascular disease, diabetes, chronic kidney disease, cancer, surgical status, primary diagnosis during current hospitalization, and preadmission use of drugs listed in Table 2. AKI, acute kidney injury; CI, confidence interval; HF, heart failure; HR, hazard ratio; ICU, intensive care unit; MI, myocardial infarction; n, number.

### Risk of myocardial infarction

The three-year cumulative risk of MI was 1.0% for patients without AKI, 1.8% for patients with AKI stage 1 and 2.3% for patients with AKI stages 2 to 3 (Figure 1 and Table 3). The adjusted HR was 1.04 (95% CI, 0.71 to 1.51) for patients with AKI stage 1 and 1.51 (95% CI, 1.05 to 2.18) for patients with AKI stages 2 to 3 (Table 3).

### Risk of stroke

Stroke risk within the first three years after hospital discharge was 0.9% for patients without AKI, 1.7% for patients with AKI stage 1, and 1.4% for patients with AKI stages 2 to 3 (Figure 1 and Table 3). The adjusted HRs were 1.10 (95% CI, 0.75 to 1.62) for patients with AKI stage 1 and 1.07 (95% CI, 0.70 to 1.65) for patients with AKI stages 2 to 3 (Table 3).

### Renal recovery

Of the 4,792 ICU patients with AKI who survived to hospital discharge, 3,888 (81.1%) recovered renal function by the time of discharge. The increased risk of HF following any stage of AKI and of MI following AKI stages 2 to 3 persisted in patients who recovered their renal function, although their risk was lower than among patients without renal recovery (Table 4).

**Table 4:** **Three-year risk of cardiovascular disease stratified by renal recovery status at hospital discharge** ^**a**^

	**Recovery (n = 3,888)**		**Nonrecovery (n = 904)**	
	**Events**	**Adjusted HR** ^**b**^	**Events**	**Adjusted HR** ^**b**^
	**n**	**(95%** **CI)**	**n**	**(95% ** **CI)**
**AKI stage 1**				
Heart failure	98	1.26 (1.00-1.60)	15	1.81 (1.07-3.07)
Myocardial infarction	35	1.05 (0.72-1.55)	3	0.79 (0.25-2.51)
Stroke	32	1.142 (0.77-1.70)	3	0.98 (0.31-3.10)
**AKI stages 2-3**				
Heart failure	62	1.50 (1.13-1.98)	30	1.42 (0.96-2.10)
Myocardial infarction	23	1.31 (0.83-2.07)	17	1.97 (1.17-3.32)
Stroke	19	1.16 (0.71-1.90)	7	0.94 (0.43-2.04)

^a^Compared to ICU patients without AKI; ^b^adjusted for age, gender, other ischemic heart diseases, other cerebrovascular diseases, hypertension, peripheral vascular disease, diabetes, chronic kidney disease, cancer, surgical status, primary diagnosis during current hospitalization, and preadmission use of drugs listed in Table 2.

AKI, acute kidney injury; CI, confidence interval; HR, hazard ratio; ICU, intensive care unit; n, number.

### Sensitivity analysis

The associations between AKI and heart failure, MI and stroke were similar after imputation of AKI level in patients without a measured plasma creatinine upon or after ICU admission. Please see Additional file 3.

## Discussion

In this population-based study of more than 21,000 ICU survivors, patients with AKI stages 2 to 3 were at 50% increased risk of HF and MI in the three-year follow-up period. Even AKI stage 1 was associated with increased risk of HF. The increased risk persisted in patients who recovered their renal function by the time of hospital discharge, but was less pronounced than the risk among patients without recovery of renal function. No associations were found between AKI and stroke or between AKI stage 1 and MI.

### Other studies

Our study is the first to examine the impact of AKI on first-time hospitalization for HF, MI, and stroke following an ICU stay. Previous studies primarily examined the impact on subsequent cardiovascular disease of AKI secondary to HF, MI or coronary intervention [13-18], and none focused on first-time cardiovascular events [13-19].

Five earlier studies examined the impact of AKI on HF [13-16,19]. Similar to our findings among ICU patients with AKI, they reported that AKI, as a complication of coronary angiography [13], MI [14], coronary arterial bypass grafting (CABG) surgery [15], HF [16], and HIV [19] increased the risk of hospitalization for subsequent HF. In a Canadian cohort of 14,782 patients who underwent coronary angiography, procedures complicated by AKI were associated with increased risk of subsequent hospital admission for HF after median follow-up of 20 months. The adjusted HRs ranged from 1.48 (95% CI, 1.16 to 1.91) for patients with AKI stage 1 to 2.17 (95% CI, 1.49 to 3.15) for patients with AKI stages 2 to 3 [13]. Similarly, a single-center Israeli study of 1,957 patients admitted for ST-elevation MI found that during median follow-up of 36 months AKI was associated with subsequent risk of HF among patients who survived until hospital discharge. This study also found that the association persisted in patients who recovered renal function before hospital discharge, which our findings confirmed [14]. A large Swedish study of 24,018 patients who underwent CABG surgery found that among 30-day survivors the adjusted HR of HF during mean follow-up of 4.1 years was 1.69 (95% CI, 1.48 to 1.94), 2.33 (95% CI, 1.69 to 3.22) and 1.87 (95% CI, 0.84 to 4.20) for AKI stages 1, 2 and 3, respectively [15]. The association between AKI and subsequent increased risk of HF was also evident in a cohort of US veterans with HIV who survived the first three months after hospital discharge. Choi *et al.* reported an adjusted HR of subsequent HF of 1.17 (95% CI, 1.34 to 2.35) in patients with stage 1 AKI, 2.11 (95% CI, 1.07 to 2.35) in patients with AKI stages 2 to 3 not requiring dialysis, and 4.20 (95% CI, 2.24 to 7.88) in patients with AKI stages 2 to 3 requiring dialysis, during mean follow-up of 5.7 years [19]. In addition, a Chinese single-center study of 1,005 patients found that patients hospitalized for HF complicated by AKI were readmitted more frequently for HF during the following year [16].

A few studies have examined risk of MI after AKI in different study populations [13,14,17,18]. Like our ICU study, they found that AKI was associated with later MI. However, these relatively small studies were hampered by imprecise risk estimates [13,14]. The study by James *et al.* found that the adjusted HRs of MI complicated by AKI stage 1 and AKI stages 2 to 3 were 1.47 (95% CI, 1.12 to 1.91) and 1.19 (95% CI, 0.70 to 2.02), respectively, compared with non-AKI patients. The Israeli study by Goldberg *et al*. found that the adjusted HR of recurrent MI after initial MI complicated by AKI was 1.6 (95% CI, 0.9 to 1.8) for patients with mild AKI who did not regain renal function by hospital discharge, 1.4 (95% CI, 0.7 to 3.0) for patients with moderate/severe AKI who did regain renal function, and 1.4 (95% CI, 0.7 to 2.8) for those with moderate/severe AKI who did not recover renal function. However, they found no increased risk of MI among patients with mild AKI who regained renal function (adjusted HR = 0.6 (95% CI, 0.2 to 1.8). In their single-center study, Lindsay *et al.* found that AKI (serum creatinine >50% over baseline) after percutaneous intervention was associated with a twofold increased risk of MI among hospital survivors (adjusted OR = 2.0; 95% CI, 1.3 to 3.2). A Danish single-center study among elective cardiac surgical patients found a nonsignificant association between AKI and increased MI risk (five-year adjusted HR = 1.5 (95% CI, 0.7 to 3.2)) [18].

To our knowledge, only two studies have examined the association between AKI and long-term (beyond 90 days) risk of stroke [13,18]. In line with our results, these studies did not find marked evidence for an association between AKI and subsequent hospitalization with stroke [13,18].

### Strengths and limitations

The main strengths of this study are its population-based design in the setting of a uniform tax-supported health care system providing equal access to health care. As well, patient follow-up was virtually complete, and data on ICU admissions were highly valid. This reduced potential selection bias.

Several limitations should be considered when interpreting our results. First, like most other studies of AKI, we lacked data on urine output and thus could not include the urine output criteria specified in the KDIGO classification. Therefore, we may have underestimated the prevalence of AKI compared to the use of both plasma creatinine and urine output criteria [40]. Second, we excluded patients without measured plasma creatinine upon or after ICU admission, which potentially could introduce selection bias. However, our results were similar after imputation of AKI level for patients without a plasma creatinine measurement. Third, despite the fact that we had access to all plasma creatinine measurements for outpatient clinic or general practitioner visits in the study area, we did not have a measured baseline creatinine for 35% of the ICU patients. We therefore estimated baseline creatinine as previously suggested [27]. This may, however, underestimate the AKI prevalence in patients without CKD [41]. In addition, even a measured plasma creatinine measurement from an outpatient clinic or a general practitioner visit, which was available in 65% of the included patients, may not truly reflect a baseline creatinine level. Fourth, our outcome assessment relies on correct coding. Previous studies have found that the positive predictive values of discharge diagnoses recorded for HF, MI, and stroke are 81% [42], 92% [43], and 84% [44], respectively. However, any misclassification is expected to be nondifferential, biasing the study toward a null result. Fifth, we had few outcomes for MI and stroke, resulting in imprecise estimates. Six, patients with coexisting HF may have been undiagnosed due to this disease’s asymptomatic nature in its early phase. The association between AKI and apparent subsequent HF therefore could be partly due to reverse causation. Finally, although we controlled for potential confounders using highly valid data on known comorbidities and drug use, our results may have been affected by unmeasured or residual confounding, such as undiagnosed cardiac dysfunction and severity of various comorbidities.

## Conclusions

AKI stages 2 to 3 in surviving ICU patients is associated with increased risk of HF and MI up to three years after hospital discharge. The association of HF was evident even among patients with AKI stage 1. These associations were less pronounced but persisted among patients who had recovered renal function by hospital discharge. This may suggest a need for systematic follow-up of patients discharged after an AKI episode, especially those who do not regain their renal function before discharge.

## Key messages

- Acute kidney injury is associated with increased risk of heart failure and myocardial infarction, but not stroke.
- The increased risk of heart failure and myocardial infarction persisted among patients who had recovered renal function by hospital discharge.

## Additional files

Additional file 1:
**Relevant codes used in the current study.**
Additional file 2:
**Characteristics of patients with missing plasma creatinine measurements, patients without AKI, and with AKI.**
Additional file 3:
**Adjusted HR of cardiovascular diseases after multiple imputation of AKI stages in patients without measured plasma creatinine from ICU admission to hospital discharge.**

## Abbreviations

AKI: acute kidney injury
ATC: anatomic therapeutic chemical classification system
CABG: coronary arterial bypass grafting
CI: confidence interval
CKD: chronic kidney disease
DNRP: Danish National Registry of Patients
eGFR: estimated glomerular filtration rate
HF: heart failure
HIV: human immunodeficiency virus
HR: hazard ratio
ICD-10: International Classification of Disease, 10^th^ revision
ICU: intensive care unit
IQR: interquartile range
KDIGO: Kidney Disease Improving Global Outcome
MDRD: Modification of Diet in Renal Disease
MI: myocardial infarction
NSAIDS: nonsteroidal anti-inflammatory drugs
