# Supplementary material for: Three-year risk of cardiovascular disease among intensive care patients with acute kidney injury: a population-based cohort study
Source: Crit Care. 2014 Oct 14;18(5):492. doi: 10.1186/s13054-014-0492-2 (PMC4197334; doi:10.1186/s13054-014-0492-2)
Supplement: Additional file 1: — Relevant codes used in the current study. [file 13054_2014_492_MOESM1_ESM.pdf]

Relevant codes used in the current study

Description	Codes
**Diseases (ICD-10)**	
Myocardial infarction	I21
Stroke	I61,I63, I64
Heart failure	I50, I11.0, I13.0, I13.2
Ischemic heart disease (except MI)	I20-I25 (except I21)
Cerebrovascular disease (except stroke)	I60-I69 (except I63-I64)
Diabetes	E10E14, O24 (except O24.4), G63.2, H36.0, N08.3
Peripheral vascular disease	I70, I71, I72, I73, I74, I77
Hypertension	I10-I15
Cancer	C00-C96
**Primary diagnosis during current hospitalization (ICD-10)**	
Infectious diseases	A00-B99, G00-G07, I00-I02, I30.1, I32.0, I33, I38, I40.0, J00-J06, J36, J39.0, J10-J22, J85.1, J86, K35, K37, K57.0, K57.2, K57.4, K57.8, K61, K63.0, K65.0, K65.9, K67, K75.0, K75.1, K80.0, K80.3, K80.4, K81.0, K81.9, K83.0, L00-L03, L05-L08, M00, M01, M86, N10, N12, N15.1, N30, N39.0, N41, N45, N70-N77
Cancer	C00-D89
Endocrine diseases	E00-E90
Cardiovascular diseases	I00-I99 without I00-I02, I30.1, I32.0, I33, I38, I40.0
Respiratory diseases	J00-J99 without J00-J06, J10-J22, , J36, J39.0, J85.1, J86
Gastrointestinal or liver diseases	K00-K99 without K35, K37, K57.0, K57.2, K57.4, K57.8, K61, K63.0, K65.0, K65.9, K67, K75.0, K75.1, K80.0, K80.3, K80.4, K81.0, K81.9, K83.0.
Trauma or poisoning	S00-S99, T00-T97
Other	All codes not included in other categories
**Treatments (Procedure codes)**	
Intensive therapy	NABE, NABB
Acute dialysis	BJFD0
Mechanical ventilation	BGDA0
Treatment with inotropes or vasopressors	BFHC92, BFHC93, BFHC95
**Laboratory data (NPU- and local laboratory codes**	
Plasma Creatinine	NPU18016, NPU01807, NPU04998. ASS00356, ASS00354, ASS00355
Hemoglobin A1c	NPU03835, NPU02307, NPU27300, DNK35249, AAB00092, AAB00091, AAA00740, AAB00061
**Drugs (ATC-codes)**	
Diuretics	C03
Beta blockers	C07
Calcium channel antagonists	C08
ACE inhibitors/AT2 antagonist	C09A, C09BA, C09BB, C09CA, C09DA, C09DB
Acetylsalicylic acid	B01AC06;
Nitrates	C01DA
Statins	C10AA
NSAIDS	M01A
**Surgical procedures (NCSP codes)**	
Cardiac surgery	KF
Non-cardiac surgery	All codes include in other categories

ACE, angiotensin converting enzyme; ATC, Anatomical Therapeutic Chemical; AT2, angiotensin 2; ICD-10, International Classification of Diseases, 10th revision; NSCP, Nordic Medico-Statistical Classification of Surgical Procedures; NPU, Nomenclature, Properties and Units in Laboratory Medicine; NSAIDSnon-steroidal anti-inflammatory drugs
