# Supplementary material for: Three-year risk of cardiovascular disease among intensive care patients with acute kidney injury: a population-based cohort study
Source: Crit Care. 2014 Oct 14;18(5):492. doi: 10.1186/s13054-014-0492-2 (PMC4197334; doi:10.1186/s13054-014-0492-2)
Supplement: Additional file 2: — Characteristics of patients with missing plasma creatinine measurements, patients without AKI, and with AKI. [file 13054_2014_492_MOESM2_ESM.pdf]

Characteristics of patients with missing plasma creatinine measurements, patients without AKI, and with AKI

	Missing P-creatinine n = 1,846	Without AKI n = 16,764	AKI stage 1 n = 2,666	AKI stages 2-3 n = 2,126
**Age**				
Age, median (IQR)	39 (28–56)	57 (39–69)	68 (58–77)	67 (56–75)
**Gender**				
Female	1,041 (56.4)	7,788 (46.5)	1,031 (38.7)	944 (44.4)
Male	805 (43.6)	8,976 (53.5)	1,635 (61.3)	1,182 (55.6)
**Comorbidity**				
Ischemic heart disease[a]	61 (3.3)	1,863 (11.1)	513 (19.2)	244 (11.5)
Cerebrovascular disease[b]	26 (1.4)	537 (3.2)	124 (4.7)	86 (4.0)
Diabetes	74 (4.0)	1490 (8.9)	460 (17.3)	433 (20.4)
Chronic kidney disease	69 (3.7)	1,063 (6.3)	502 (18.8)	338 (15.9)
Hypertension	131 (7.1)	2,216 (13.2)	664 (24.9)	511 (24.0)
Peripheral vascular disease	26 (1.4)	721 (4.3)	266 (10.0)	202 (9.5)
Cancer	110 (6.0)	2,282 (13.6)	522 (19.6)	375 (17.6)
**Surgical Status**				
No surgery	589 (31.9)	6,455 (38.5)	662 (24.8)	793 (37.3)
Surgery				
Acute cardiac surgery	3 (0.2)	195 (1.2)	88 (3.3)	59 (2.8)
Acute non-cardiac surgery	867 (47.0)	5,558 (33.2)	834 (31.3)	764 (35.9)
Elective cardiac surgery	6 (0.3)	1,701 (10.1)	528 (19.8)	169 (7.9)
Elective non-cardiac surgery	381 (20.6)	2,855 (17.0)	554 (20.8)	341 (16.0)
**Preadmission drug use**				
ACE inhibitors/AT2 antagonists	154 (8.4)	2,677 (16.0)	763 (28.6)	698 (32.8)
Beta blockers	165 (8.9)	2,318 (13.8)	667 (25.0)	487 (22.9)
Calcium channel antagonists	78 (4.2)	1587 (9.5)	483 (18.1)	401 (18.9)
Acetylsalicylic acid	281 (15.2)	5,286 (31.5)	1,362 (51.1)	1,005 (47.3)
Diuretics	83 (4.5)	1348 (8.0)	357 (13.4)	272 (12.8)
Nitrates	17 (0.9)	756 (4.5)	236 (8.9)	83 (3.9)
Statins	99 (5.4)	2,533 (15.1)	671 (25.2)	441 (20.7)
NSAIDs	252 (13.6)	2,486 (14.8)	430 (16.1)	436 (20.5)
**Primary diagnosis during current hospitalization**				
Infectious diseases	257 (13.9)	1,673 (10.0)	276 (10.4)	434 (20.4)
Endocrine diseases	50 (2.7)	302 (1.8)	69 (2.6)	99 (4.7)
Cardiovascular diseases	140 (7.6)	2,910 (17.4)	873 (32.7)	390 (18.3)
Respiratory diseases	53 (2.9)	741 (4.4)	118 (4.4)	108 (5.1)
Gastrointestinal or liver diseases	133 (7.2)	1,564 (9.3)	287 (10.8)	303 (14.3)
Cancer or other neoplasm	98 (5.3)	2,463 (14.7)	471 (17.7)	304 (14.3)
Trauma or poisoning	371 (20.1)	4,198 (25.0)	282 (10.6)	190 (8.9)
Other	744 (40.3)	2,913 (17.4)	290 (10.9)	298 (14.0)
**Laboratory information**				
Measured baseline creatinine	782 (42.4)	10,213 (60.9)	2143 (80.4)	1,610 (75.7)
Maximum creatinine during admission[d], µmol/L, mean (IQR)	NA	74 (62–88)	123 (101–149)	254 (180–397)
**ICU treatments**				
Mechanical ventilation	52 (2.8)	4,362 (26.0)	1,297 (48.6)	1,126 (53.0)
Inotropes/vasopressors	21(1.1)	3,083 (18.4)	1,158 (43.4)	1,155 (52.9)
**Length of admission**				
In-hospital days,[e] median (IQR)	2 (1–4)	8 (3–15)	15 (10–28)	23 (12–45)

Values are expressed as number (percentage) unless otherwise indicated.
[a] Patients with a previous diagnosis of myocardial infarction were not included in the study.
[b]Patients with a previous diagnosis of stroke were not included in the study.
ACE, angiotensin converting enzyme; AKI, acute kidney injury; AT2, angiotensin 2; CI, confidence interval; ICU, intensive care unit; IQR, inter-quartile range; NA, not available; NSAIDS, non-steroidal anti-inflammatory drugs
