# Supplementary material for: Three-year risk of cardiovascular disease among intensive care patients with acute kidney injury: a population-based cohort study
Source: Crit Care. 2014 Oct 14;18(5):492. doi: 10.1186/s13054-014-0492-2 (PMC4197334; doi:10.1186/s13054-014-0492-2)
Supplement: Additional file 3: — Adjusted HR of cardiovascular diseases after multiple imputation of AKI stages in patients without measured plasma creatinine from ICU admission to hospital discharge. [file 13054_2014_492_MOESM3_ESM.pdf]

Adjusted HR of cardiovascular diseases after multiple imputation of AKI stages in patients without measured plasma creatinine from ICU admission to hospital discharge[a]

	AKI stage 1<br>Adjusted HR (95% CI)	AKI stages 2–3<br>Adjusted HR (95% CI)
Heart failure	1.31 (1.00–1.64)	1.44 (1.13–1.82)
MI	1.08 (0.74–1.57)	1.56 (1.08–2.24)
Stroke	1.12 (0.71–1.68)	1.09 (0.71–1.68)

[a] Compared with patients without AKI.

AKI, acute kidney injury; CI, confidence interval; HR, hazard ratio; ICU, intensive care unit; MI, myocardial infarction.
